# Supplementary material for: No-boarding buses: Synchronisation for efficiency
Source: PLoS One. 2020 Mar 23;15(3):e0230377. doi: 10.1371/journal.pone.0230377 (PMC7089533; doi:10.1371/journal.pone.0230377)
Supplement: S1 Data — Live data on the NTU campus shuttle buses can be found here: https://baseride.com/maps/public/ntu/. (DOCX) [file pone.0230377.s002.docx]

**NTU campus shuttle buses**

Live data on the NTU campus shuttle buses can be found here: https://baseride.com/maps/public/ntu/
